# Sleep Disturbance and Its Association With Sluggish Cognitive Tempo and Attention in Pediatric Brain Tumor Survivors

**DOI:** 10.3389/fnins.2022.918800

**Published:** 2022-06-23

**Authors:** Ineke M. Olsthoorn, Alice Ann Holland, Raymond C. Hawkins, Allen E. Cornelius, Muhammad Usman Baig, Grace Yang, Daniel C. Holland, Wafik Zaky, Peter L. Stavinoha

**Affiliations:** ^1^Department of Neurology, McGovern Medical School, University of Texas Health Science Center at Houston (UT Health), Houston, TX, United States; ^2^Department of Psychiatry, University of Texas Southwestern Medical School, Dallas, TX, United States; ^3^Department of Psychiatry, Children’s Medical Center of Dallas, Dallas, TX, United States; ^4^School of Psychology, Fielding Graduate University, Santa Barbara, CA, United States; ^5^Department of Pediatrics, University of Texas MD Anderson Cancer Center, Houston, TX, United States

**Keywords:** sleep, sluggish cognitive tempo, attention, executive functioning, pediatric, brain tumor survivor, cancer

## Abstract

**Background:**

Pediatric brain tumor (PBT) survivors are at risk for developing sleep disturbances. While in other pediatric populations sleep disturbance has been associated with worse cognitive functioning, it is unclear to what extent this relationship generalizes to PBT survivors. The aim of the current study was to assess the relationship between sleep disturbance and aspects of cognition, including sluggish cognitive tempo (SCT) as well as attention and working memory.

**Materials and Methods:**

Eighty-three PBT survivors 6–18 years of age who were at least 3 months post-treatment were included in the present cross-sectional study. Level of sleep disturbance was measured as a composite score reflecting various sleep problems as rated by caregivers. Cognitive measures included caregiver-ratings of sluggish cognitive tempo and attention problems, as well as performance-based cognitive measures assessing attention and executive functioning. Hierarchical regression analysis was used to assess associations between sleep and cognition.

**Results:**

Of all caregivers, 32.5% reported one or more sleep disturbances as “very/often true” and over 68% of caregivers rated at least one sleep-related item as “somewhat true.” Of all cognitive variables, scores were most frequently impaired for SCT (30%). A higher level of sleep disturbance was associated with worse SCT and parent-rated attention problems. Associations between sleep and performance-based cognitive measures assessing attention and working memory were not statistically significant.

**Conclusion:**

Findings of the current study highlight the importance of further investigation into the relationship between sleep and cognition in PBT survivors, which may assist efforts to maximize cognitive outcome and health-related quality of life in PBT survivors. The current study additionally suggests further investigation of SCT in this population is warranted, as it may be more sensitive to detecting possible associations with sleep disturbance relative to discrete measures that assess cognitive performance under ideal circumstances.

## Introduction

As survival rates for children and adolescents diagnosed with a brain tumor have improved, the importance of maximizing health-related quality of life (HRQOL) in pediatric brain tumor (PBT) survivors has been increasingly recognized (Burns et al., 2016; Netson et al., 2016). Cognitive dysfunction has been related to worse quality of life in PBT survivors, and understanding risk factors for cognitive dysfunction may inform prevention and treatment recommendations to maximize cognitive outcomes. Many factors have been found to be associated with cognitive impairments in PBT survivors, including—both collectively and individually—the tumor, secondary neurological complications, and treatment-related factors such as radiation therapy, chemotherapy, and surgery (Hardy et al., 2008; Tonning Olsson et al., 2014; Taiwo et al., 2017; Ikonomidou, 2018).

A less extensively researched factor that may impact cognitive functioning and HRQOL in PBT survivors is suboptimal sleep (Daniel et al., 2016; Merz and Tomfohr-Madsen, 2016), especially since sleep disruption has been associated with reduced HRQOL in broad samples of pediatric cancer patients and survivors (Kaleyias et al., 2012; Steur et al., 2016). In healthy pediatric populations, it has been shown that suboptimal sleep may negatively affect brain maturation and the associated development of cognitive functions (Peirano and Algarín, 2007; Astill et al., 2012). Sleep disturbances have been associated with changes in networks related to working memory and attention, including the frontal-parietal and default mode networks (de Havas et al., 2012; Nie et al., 2015; Yeo et al., 2015; Krause et al., 2017; Tashjian et al., 2018). Additionally, disrupted sleep may result in changes in motivation and effort perception which, in turn, may affect performance (Robert and Hockey, 1997; Hockey, 2011, 2013; Monk, 2012; Massar et al., 2019).

Relevant to PBT survivors, it is noteworthy that in healthy children and adolescents, shorter sleep duration has been particularly associated with worse performance on cognitive tests assessing working memory, processing speed, and sustained attention (Astill et al., 2012; Vriend et al., 2013; Louca and Short, 2014)—functions which are commonly compromised in PBT survivors (Ullrich and Embry, 2012; Wolfe et al., 2012; Hoang et al., 2014). Furthermore, it is known that PBT survivors have an elevated risk for sleep difficulties (Gapstur et al., 2009; Rosen and Brand, 2011; Nolan et al., 2013; Brimeyer et al., 2016; Desaulniers et al., 2018). Estimates of the prevalence of sleep problems in PBT survivors have ranged from 20 to 82% (Brimeyer et al., 2016; Pilotto et al., 2018; van Kooten et al., 2019), as compared to rates of 12–25% in healthy populations (Melendres et al., 2004; van Litsenburg et al., 2010). The wide range of estimates for PBT survivors reflects that sleep disturbances are often described as general symptoms rather than specific to criteria for a sleep disorder (Otte et al., 2015; Daniel et al., 2020). Sleep disturbances can persist into adulthood for pediatric cancer survivors (Zhou and Recklitis, 2014; Daniel et al., 2020). In fact, excessive daytime sleepiness has been found to be more common in survivors of pediatric brain tumors as compared to survivors of other pediatric cancers (Verberne et al., 2012).

A range of sleep disturbances have been observed in pediatric cancer patients and survivors, including insomnia, excessive daytime sleepiness, poor sleep quality, circadian rhythm disorders, parasomnias, and sleep-disordered breathing (van Someren et al., 2004; Rosen and Brand, 2011; Nolan et al., 2013; Zhou and Recklitis, 2014; Sheikh et al., 2021). Various mechanisms have been proposed to explain elevated rates of sleep disturbances in children diagnosed with cancer (Daniel et al., 2016; Merz and Tomfohr-Madsen, 2016). Potential contributing factors are those specific to the disease and its treatment (e.g., damage to brain areas involved in sleep; Mandrell et al., 2020; Klages et al., 2021; Sheikh et al., 2021), environmental factors (e.g., sleep disruption due to hospitalizations or spending excessive time in bed; Hinds et al., 2007; Linder and Christian, 2012; Lee et al., 2017), family factors (e.g., increased distress or conflict due to altered family dynamics; Pai et al., 2007; El-Sheikh et al., 2012; Marcus, 2012; Pollock et al., 2013; Wiener et al., 2017), psychological factors specific to the child (e.g., traumatic stress, anxiety or depression; Shah et al., 2015; McDonnell et al., 2017; Kim et al., 2020), and reduced physical activity (Orsey et al., 2013; Antwi et al., 2019).

One construct that has been associated with sleep disturbances in both healthy adolescents as well as children and adolescents with neurodevelopmental conditions (such as ADHD) is sluggish cognitive tempo (SCT; e.g., Mayes et al., 2021; Fredrick et al., 2022), which refers to a set of symptoms that includes mental fogginess and confusion, slowed behavior/thinking, daydreaming, feelings of confusion, and low motivation (Becker et al., 2016). However, SCT does not merely reflect a cluster of individually measurable cognitive functions (Becker, 2021), and its empirical differentiation from constructs such as internalizing emotional disorders (e.g., anxiety, depression) and ADHD-related symptoms (e.g., inattention and hyperactivity) suggests it should be seen as a distinct entity (Smith et al., 2019). SCT has also been found to be distinct from excessive daytime sleepiness (Langberg et al., 2014; Smith et al., 2019), and in fact has been shown to mediate the relationship between sleep disturbance and academic performance in a sample of healthy children (O’Hare et al., 2021). In recent years, SCT has been associated with deficits in various cognitive functions that are commonly observed in the PBT population—namely, working memory, cognitive speed, attention, and executive function (Becker et al., 2016; Stavinoha et al., 2018; Kofler et al., 2019; Rey-Casserly and Diver, 2019). Researchers have additionally noted that the concept of SCT offers a unique framework for understanding the neurocognitive challenges faced by PBT survivors (Kahalley et al., 2011; Willard et al., 2013; Peterson et al., 2021). However, to our knowledge, SCT has not explicitly been studied in relation to sleep disturbances in PBT survivors.

Regarding cognitive functions commonly associated with SCT in PBT survivors, studies of children with mixed neurological conditions have demonstrated that worse sleep quality relates to worse performance on measures of attention, working memory, and cognitive efficiency (McCann et al., 2018a,b). In adult oncology populations, researchers have also observed associations between sleep and self-rated cognitive impairment, memory, and attention (Chen et al., 2012; Jean-Pierre et al., 2015; Myers et al., 2015; von Ah and Tallman, 2015; Janelsins et al., 2016), as well as associations between sleep and performance on measures of verbal and executive functioning (Caplette-Gingras et al., 2013; Hartman et al., 2015). Furthermore, in adult survivors of pediatric cancer, insomnia has been related to worse performance on measures of executive functioning, attention, processing speed, and other cognitive functions (Tonning Olsson et al., 2020).

Nonetheless, scant research has investigated sleep and cognition in survivors of pediatric cancer who have not yet reached adulthood. Associations between sleep disturbance and performance on executive functioning and processing speed measures have been observed in female survivors of acute lymphoblastic leukemia (Cheung et al., 2017), and sleep disturbances have been associated with worse executive functioning in mixed PBT samples (van Kooten et al., 2019). Conversely, studies of small samples of children and adolescents with craniopharyngioma have not demonstrated a relationship between excessive daytime sleepiness and cognitive outcomes such as attention and executive functions (Jacola et al., 2016). A recent study identified a relationship between cognitive speed and fatigue among pediatric survivors of posterior fossa tumors (Levitch et al., 2022), but this investigation did not isolate sleep disturbances as separate from fatigue. Sleep and fatigue are distinct concepts in pediatric oncology, with fatigue defined more as a lack of energy and feeling of exhaustion not necessarily associated with sleepiness (Walter et al., 2015).

The need for more sleep research in pediatric cancer populations, including investigations of the role of sleep in neurocognitive outcomes, has been highlighted in a recent position paper by the International Psycho-Oncology Society Pediatrics Special Interest Group (Daniel et al., 2020). Sleep represents a potentially modifiable target for intervention to possibly improve aspects of HRQOL and related cognitive performance in domains that are particularly vulnerable after treatment for PBT. Given the relationships that have been identified between sleep and cognition in healthy children and adolescents, adult cancer survivors, and pediatric samples with various neurologic conditions, the purpose of the current study was to first examine the association between sleep disturbance and sluggish cognitive tempo (SCT) in a clinical sample of PBT survivors. Because SCT is associated with cognitive vulnerabilities that commonly occur in PBT survivors, a secondary aim was to examine the association between sleep disturbance and the specific cognitive functions of working memory, reaction speed, and attention. We hypothesized that sleep disturbance would be associated with greater SCT, and that performance on individual cognitive domains of attention, working memory, and reaction time would be poorer as sleep disturbance increased.

## Materials and Methods

### Participants and Procedure

The data for the current cross-sectional, retrospective study were obtained from a referred sample of PBT survivors seen for neuropsychological evaluation between January 1, 2005, and July 24, 2019, at the University of Texas MD Anderson Cancer Center, Department of Pediatrics, in Houston, TX, United States. The study was approved by Institutional Review Board (IRB) of the University of Texas MD Anderson Cancer Center. In addition to the administration of cognitive tests to PBT survivors as part of clinical evaluations, caregivers completed standardized questionnaires regarding their child. All included participants had a history of a brain tumor and had completed treatment. PBT survivors were defined as having completed all tumor-directed active treatment (i.e., surgery, chemotherapy, radiation) at least 3 months prior to evaluation, a cutoff that has been used previously (Barrera et al., 2018). Other inclusion criteria included an age range between 6:0 and 18:11 years (to align with the measures of interest) and completion of neuropsychological testing in either English or Spanish. Although participants were not systematically screened for other neurological or neurodevelopmental conditions during the clinical assessment, children who were determined to have a developmental delay not explained by their brain tumor (e.g., predating tumor diagnosis) based on a review of medical records by a neuropsychologist were excluded from analysis. Of 90 identified potential participants, a total of 83 met inclusion criteria. Demographic and medical characteristics of the sample are summarized in Table 1. The age of the participants at the time of neuropsychological evaluation ranged from 6 years, 4 months to 18 years, 9 months (*M* = 12.48 years, *SD* = 3.12 years).

**TABLE 1:** Demographic and medical characteristics of participants.

	Min.	Max.	*M*	*SD*
Age at evaluation	6.33	18.75	12.48	3.11
Age at diagnosis	0.16	16.75	7.56	4.44
Years of treatment	0.42	15.17	3.53	2.96
General cognitive ability	42	127	96.24	15.46
Disease burden (Neurological Predictor Scale)	1	11	4.76	2.22

			***n***	**%**

**Gender**				
Male			35	42.2
Female			48	57.8
**Ethnicity**				
Caucasian			48	57.8
Black			6	7.2
Hispanic/Latino			22	26.5
Asian/Asian American			5	6.0
Hawaiian			1	1.2
Unknown			1	1.2
**Socioeconomic status**				
I–High			15	18.1
II			19	22.9
III			25	30.1
IV			10	12.0
V–Low			9	10.8
Unknown			5	6.0
**Tumor type**				
Medulloblastoma			10	12.0
Juvenile pilocytic astrocytoma/other astrocytoma			24	28.9
Ependymoma			7	8.4
Glioma			7	8.4
Germ cell tumor			7	8.4
Craniopharyngioma			5	6.0
Retinoblastoma			4	4.8
Optic glioma			3	3.6
Primitive neuro-ectodermal tumor (PNET)			2	2.4
Mixed or other tumor			14	16.9
**Treatment^a^**				
Radiation therapy			59	71.1
Chemotherapy			47	56.6
Surgery			70	84.3

*N = 83.*

*^a^Reflects the number and percentage of participants who received specific cancer treatments per medical record.*

### Measures

#### Child Behavior Checklist Sleep Composite

The Child Behavior Checklist [CBCL/6-18; (Achenbach and Rescorla, 2001)] was used to measure parent-reported sleep disturbance. The CBCL is a rating scale of behavioral functioning with well-established psychometric properties in clinical, non-clinical, cross-cultural, and Spanish speaking populations (e.g., Achenbach and Rescorla, 2001; Ivanova et al., 2007; Rescorla et al., 2007; Lubke et al., 2009). Although this measure was not originally designed with a specific scale measuring sleep functioning, a number of items describe aspects of sleep, reflecting the disturbances “nightmare” (item 47), “tired” (item 54), “sleeps less” (item 76), “sleeps more” (item 77), “sleep walk” (item 92), “sleep problem” (item 100), and “wets bed” (item 108). A summed CBCL sleep composite score has been previously utilized to measure sleep in pediatric samples (Gregory et al., 2011; Beebe et al., 2014; Becker et al., 2015b), which will be referred to in the current study as the CBCL Sleep Composite. All items are rated on a 3-point scale (“not true,” “somewhat/sometimes true,” and “very true/often true”) as occurring “now or in the past 6 months,” with higher scores reflecting a higher level of sleep disturbance (Achenbach and Rescorla, 2001; Becker et al., 2015b).

Several validation studies of the CBCL Sleep Composite provide support for the convergent and discriminant validity of the CBCL Sleep Composite as a screening measure for sleep disturbance in archival studies (e.g., Gregory et al., 2011; Becker et al., 2015b; Murray et al., 2016; Rondon et al., 2020). The CBCL Sleep Composite has been used in a range of studies investigating the relationship between sleep disturbance and clinical variables, including cognition (e.g., Murray et al., 2016; Hambrick et al., 2018; Rondon et al., 2020).

The present study used the CBCL Sleep Composite with six items—in the current investigation referred to as the CBCL Sleep Composite-6—based on an assessment of the internal consistency of the full scale seven-item scale (i.e., CBCL Sleep Composite-7) as compared to the internal consistency of the CBCL Sleep Composite-6. First, a reliability analysis was performed to determine the internal consistency of the CBCL Sleep Composite-7, as used in prior research (e.g., Beebe et al., 2014). The overall scale’s internal consistency, Cronbach’s α = 0.58, was below the recommended minimum cutoff value of α = 0.60 (Nunnally and Bernstein, 1994). The correlation between the item “wets bed” and the total CBCL Sleep Composite-7 score was inverse (*r* = −0.03) and substantially lower than the recommended 0.30 (Field, 2009). The extent to which bed wetting stems from sleep disturbance is not well understood (Al-Omar et al., 2014), and in the present study, the greatest increase in alpha came from deleting this item.

A second reliability analysis was conducted to determine the internal consistency of the CBCL Sleep Composite-6, which revealed fair internal consistency for the CBCL Sleep Composite-6, with Cronbach’s α = 0.62, similar to the internal consistency observed in several prior studies using this 6-item scale (Becker et al., 2015b; Rondon et al., 2018). None of the remaining items were indicated to increase Cronbach’s α further if deleted. While the items “sleeps less,” “sleeps more,” and “sleep walk” remained relatively weakly correlated with the overall CBCL Sleep Composite-6 score (i.e., *r*’s = 0.250–0.291), this was unsurprising as the CBCL Sleep Composite-6 scale includes various sleep difficulties that are not necessarily expected to co-exist (Gregory and O’Connor, 2002). Based on these results, the CBCL Sleep Composite-6 scale was selected as the primary measure of sleep disturbance in the current study.

#### Child Behavior Checklist Sluggish Cognitive Tempo and Attention Problems Subscales

The CBCL for ages 6–18 years was used to assess sluggish cognitive tempo and attention (Achenbach and Rescorla, 2001). The CBCL includes a specific Sluggish Cognitive Tempo subscale that has been previously used to assess SCT in PBT survivors (e.g., Willard et al., 2013) and was the primary measure of interest. The CBCL SCT subscale yields an age/gender normed T-score (*M* = 50, *SD* = 10), with higher scores representative of poorer functioning.

The CBCL Attention Problems subscale also yields an age/gender normed T-score (*M* = 50, *SD* = 10), with higher scores representative of poorer functioning. As compared to clinic-based discrete measures of cognitive functions such as attention, the CBCL Attention Problems subscale provides information based on caregiver/parent observation over a long period of time in the child’s everyday environment.

The combination of the CBCL SCT and Attention Problems subscales has been utilized previously investigating aspects of attention and SCT in mixed cancers, including PBT survivors (Willard et al., 2013).

#### Wechsler Digit Span Forward and Backward

Clinical assessment of attention and working memory included the Digit Span subtest of the Wechsler Intelligence Scale for Children, Fourth Edition (WISC-IV; Wechsler, 2003), either the English or the Spanish version of the Wechsler Intelligence Scale for Children, Fifth Edition (WISC-V; Wechsler, 2003, 2014), or the Wechsler Adult Intelligence Scale, Fourth Edition (WAIS-IV; Wechsler, 2008), based on each participant’s age. The WISC-IV, WISC-V, and WAIS-IV have been combined in previous research investigating the relationship between sleep and cognition (e.g., McCurdy et al., 2016; Sali et al., 2018; Calhoun et al., 2019). The subtests Digit Span Forward (DSF) and Digit Span Backward (DSB) were included. While DSF involves listening to and repeating sequences of numbers in the same order, DSB requires listening to and repeating sequences of numbers in the reverse order, with comparable instructions between the WISC-IV, WISC-V, and WAIS-IV versions (Wechsler, 2003, 2014; Calhoun et al., 2019). Norm-referenced scaled scores (*M* = 10, *SD* = 3) for the total number of correctly repeated sequences were used as an outcome measure, with higher scores reflecting a better performance. The WISC-V DSF and DSB split-half reliabilities are good (i.e., *r* = 0.81 and *r* = 0.80, respectively). WISC-IV, WISC-V, and WAIS-IV DSF and DSB test-retest reliabilities are adequate to good (i.e., corrected *r* = 0.74–0.82; Wechsler, 2003, 2008, 2014). The DSF and DSB subtests have been used previously in studies investigating the relationship between sleep and cognition (McCann et al., 2018a,b).

#### Conners Continuous Performance Test, Second Edition

The Conners Continuous Performance Test, Second Edition (CPT-II; Conners, 2000) is a computer-administered task measuring aspects of attention and reaction time. The test takes approximately 14 min to complete and requires the test-taker to press a key as quickly as possible after a letter appears on the screen, while refraining from pressing the key when the letter is an “X.” Split-half reliabilities are adequate, ranging between *r* = 0.66 and 0.95, and test-retest reliabilities are excellent for individuals in a neurological assessment setting. The test can differentiate between clinical and non-clinical groups (Homack and Riccio, 2006) and has been used before in studies investigating the relationship between cognition and sleep (Kuula et al., 2015). Omissions (i.e., missed targets), Hit Reaction Time (Hit RT), and Commissions were used to measure visual sustained attention and response speed.

#### Demographic and Control Variables

##### Disease Burden

In all analyses investigating the association between sleep disturbance and cognitive functioning, disease burden was included as a control variable, as a higher impact of disease and treatment factors has been related to lower cognitive functioning in PBT survivors (e.g., Taiwo et al., 2017), and is hypothesized to negatively affect sleep (Merz and Tomfohr-Madsen, 2016). Thus, when not controlled for disease burden, a relationship between sleep disturbance and cognition could merely reflect the impact of the disease and treatment. The Neurological Predictor Scale (Micklewright et al., 2008) was used to quantify neuro-oncological risk factors, as this measure provides a single, cumulative estimate of the exposure to tumor- and treatment-related risk factors (Micklewright et al., 2008). The NPS consists of four items, which represent various sources of neuro-oncological risk including tumor-related complications (e.g., the presence of a hormone deficiency, hydrocephalus, or antiepileptic medication), surgical events, radiation therapy, and chemotherapy. Total raw scores have been analyzed as a continuous variable in prior studies (Micklewright et al., 2008; Taiwo et al., 2017) and range from 0 to 16, with higher values reflecting a higher level of disease burden. In the present study, the scores were calculated by a physician based on information obtained from the medical records. The reliability and concurrent validity of the NPS has been demonstrated in both children and adults surviving pediatric brain tumors (Micklewright et al., 2008; Papazoglou et al., 2008; Taiwo et al., 2017).

##### Demographic Variables

Demographic variables examined in the current study were age, gender, age at diagnosis, months off treatment, socioeconomic status (SES), and general cognitive ability. SES was measured with the Hollingshead Two Factor Index of Social Position (Hollingshead, 1957), which has been used in recent studies (Allen et al., 2016; Hirakawa et al., 2019) and yields a numeric rating based on the occupation and educational attainment of caregivers. The Wechsler General Ability Index (GAI) was used to measure general cognitive ability. The GAI generally subsumes the verbal and perceptual indexes from the WISC-IV, WISC-V, and WAIS-IV, while excluding measures of working memory and cognitive efficiency. Scores are expressed in standard scores (*M* = 100, *SD* = 15), with higher scores reflective of greater cognitive ability (Wechsler, 2003, 2014).

### Statistical Analyses

Data were analyzed using SPSS Statistics Version 26. Descriptive statistical analyses were conducted to determine the characteristics of the sample, and zero-order correlations between all variables were computed in order to evaluate the relationship between all combinations of two variables. One-sample *z*-tests were performed to compare cognitive functioning scores with normative data. Data were analyzed to identify potential influential cases and violations of assumptions prior to hypothesis testing. *P*-values of <0.05 were considered to be statistically significant. Six general linear model hierarchical multiple linear regression analyses were conducted to assess the relationships between sleep disturbance and cognitive functions of interest. Disease burden (NPS score) was entered in Model I as a control variable, with the CBCL Sleep Composite-6 entered as a continuous predictor variable in Model 2. Separate regressions were conducted for each dependent measure (i.e., CBCL Sluggish Cognitive Tempo, CBCL Attention Problems, DSF, DSB, CPT-II Omissions, CPT-II Hit RT, and CPT-II Commissions). Prior to hypothesis testing, it was decided to report the results of the regression analyses without correcting for multiple testing. Given the paucity of research on the relationship between cognition and sleep in PBT survivors, missing an existing relationship between sleep and cognition (i.e., Type II error) was considered to be more harmful than reporting a false-positive (i.e., Type I error). All variables and measures used in the study are summarized in Table 2.

**TABLE 2:** Descriptive statistics for CBCL sleep items and composite.

			Frequency of item endorsement		
			
Item	*M* ± *SD*	Range^a^	Not true (0)	Somewhat/sometimes true (1)	Very/often true (2)
Nightmare		0–2	66 (79.5%)	14 (16.9%)	3 (3.6%)
Tired		0–2	56 (67.5%)	14 (16.9%)	13 (15.7%)
Sleeps less		0–2	69 (83.1%)	11 (13.3%)	3 (3.6%)
Sleeps more		0–2	55 (66.3%)	15 (18.1%)	13 (15.7%)
Sleep walk		0–1	75 (90.4%)	8 (9.6%)	0 (0.0%)
Sleep problem		0–2	60 (72.3%)	15 (18.1%)	8 (9.6%)

	***M*** **±*SD***	**Range**	**Total score of 0**	**At least one item rated as very/often true**	

CBCL Sleep Composite-6	1.89 ± 2.11	0–8	31.3%	32.5%	

*N = 83.*

*^a^Range reflects the range of observed scores. Range of possible values is 0–2 for specific items and 0–12 for CBCL Sleep Composite-6.*

## Results

### Participants

Details regarding the demographic and medical characteristics of the 83 participants are presented in Table 1. Age at evaluation ranged between 6.33 and 18.75 years (*M* = 12.48, *SD* = 3.11), and age at tumor diagnosis was 0.16–16.75 years (*M* = 7.56, *SD* = 4.44). Participants had completed treatment 0.42–15.17 years (*M* = 3.53, *SD* = 2.96) prior to evaluation. In total, 42.2% of the sample identified as male and 57.8% as female. Zero-order Pearson correlations were computed between demographic variables, disease burden, sleep disturbance, and the cognitive variables in the regression analyses. Only disease burden was significantly associated with both sleep disturbance (i.e., *r* = −0.23, *p* < 0.05) as well as cognition (i.e., DSB *r* = −0.23, *p* < 0.05; CPT-II Hit RT *r* = −0.25, *p* < 0.05; CBCL Attention Problems *r* = −0.31, *p* < 0.01). Therefore, only disease burden was included as potential confounding factor in the regression analyses in order to maximize power.

### Characteristics of Sleep Disturbance and Cognition

Details regarding CBCL Sleep Composite-6 scores and endorsement of specific items are presented in Table 2. Of all sleep-related disturbances rated to be very true, “tired” and “sleeps more” were most frequently endorsed. A total of 27 (32.5%) parents rated at least one sleep symptom as “very true” or “often true” for their child, while 26 caregivers (31.3%) rated no sleep-related symptoms. Thus, nearly two-thirds of the sample endorsed at least one sleep-related symptom.

Neuropsychological characteristics of the participants are presented in Table 3. The percentage of participants with impaired scores was highest for CBCL Sluggish Cognitive Tempo (30%), followed by CBCL Attention Problems (20.48%), DSF (17.5%), CPT-II Hit RT (16.05%), DSB (8.75%), CPT-II Omissions (7.32%), and CPT-II Commissions (4.88%). On average, participants’ scores were significantly different than the normative mean on DSF, DSB, CPT-II Hit RT, CBCL Attention Problems, and CBCL Sluggish Cognitive Tempo and all in the expected (pathological) direction for a PBT sample. Only CPT-II Omissions and CPT Commissions were not statistically significantly different from the population mean. Thus, this sample of PBT survivors on the whole appears to exhibit difficulties relative to the general population on cognitive dimensions known to be vulnerable to PBT and treatment.

**TABLE 3:** Neuropsychological characteristics.

Variable	*n*	*M*	*SD*	Min.	Max.	*z*	Cohen’s d*	*p*	% impaired**
**CBCL**									
SCT	80	58.19	8.39	50	75	7.33	0.82	<0.001	30.00
Attention problems	83	58.37	8.72	50	86	7.63	0.84	<0.001	20.48
**Wechsler**									
DSF	80	8.35	2.84	3	15	–4.92	–0.55	<0.001	17.50
DSB	80	8.97	2.78	1	16	–3.07	–0.34	<0.01	8.75
**CPT-II**									
Omissions	82	51.52	13.01	40.06	122.38	1.38	0.15	0.17	7.32
Hit RT	81	53.09	14.15	27.10	93.30	2.78	0.31	<0.01	16.05
Commissions	82	49.70	10.99	21.04	70.77	–0.91	–0.10	0.36	4.88

*As compared to the normative sample.

**Percentage of individuals with scores 1.5 standard deviation from the mean.

### Association Between Sleep Disturbance and Cognition

Prior to conducting the regression analyses, it was determined that assumptions were satisfactorily met, and no concerns were identified regarding influential cases. Notably, the percentage of missing data for each variable included in the analyses was 3.6% (*n* = 3) at most. Results of separate hierarchical multiple regression analyses examining relationships between sleep disturbance and measures of cognitive functioning after controlling for NPS score are shown in Table 4. For the primary regression analysis with CBCL Sluggish Cognitive Tempo as the dependent variable, Model 1 indicated that the variance accounted for (*R*^2^) by the control variable (i.e., disease burden) equaled 0.04, which was not significantly different from zero, *F*(1,78) = 3.49, *p* = 0.07. In Model 2, the change accounted for by the CBCL Sleep Composite-6 (Δ*R*^2^) was equal to 0.28, which was significantly different from zero, *F*(1,77) = 32.47, *p* < 0.001. Thus, 28% of the variance in Sluggish Cognitive Tempo was accounted for by the CBCL Sleep Composite-6 score.

**TABLE 4:** Hierarchical regression results for cognitive variables.

Variable	*B*	95% CI for *B*		*SE B*	β	*R* ^2^	Δ*R*^2^
		
		*LL*	*UL*				
**Dependent variable: CBCL Sluggish Cognitive Tempo**							
Step 1						0.04	0.04
Constant	61.90	57.54	66.25	2.19			
Neurological Predictor Scale	–0.77	–1.60	0.05	0.41	–0.21		
Step 2						0.33	0.28***
Constant	55.54	51.24	59.84	2.16			
Neurological Predictor Scale	–0.30	–1.01	0.42	0.36	–0.08		
CBCL Sleep Composite-6	2.16	1.40	2.91	0.38	0.55		
**Dependent variable: CBCL Attention Problems**							
Step 1						0.09	0.09**
Constant	64.12	59.78	68.46	2.18			
Neurological Predictor Scale	–1.21	–2.03	–0.38	0.42	–0.31		
Step 2						0.28	0.19***
Constant	58.78	54.23	63.33	2.29			
Neurological Predictor Scale	–0.81	–1.58	–0.05	0.38	–0.21		
CBCL Sleep Composite-6	1.83	1.03	2.63	0.40	0.44		
**Dependent variable: Digit Span Forward**							
Step 1						0.01	0.01
Constant	8.83	7.33	10.34	0.75			
Neurological Predictor Scale	–0.10	–0.39	0.19	0.15	–0.08		
Step 2						0.01	0.00
Constant	8.95	7.17	10.72	0.89			
Neurological Predictor Scale	–0.11	–0.41	–0.19	0.15	–0.09		
CBCL Sleep Composite-6	–0.04	–0.36	0.28	0.16	–0.03		
**Dependent variable: Digit Span Backward**							
Step 1						0.05*	0.05*
Constant	10.31	8.88	11.75	0.72			
Neurological Predictor Scale	–0.29	–0.56	–0.01	0.14	–0.23		
Step 2						0.06*	0.01
Constant	9.99	8.30	11.68	0.85			
Neurological Predictor Scale	–0.26	–0.55	0.03	0.14	–0.21		
CBCL Sleep Composite-6	0.11	–0.19	0.41	0.15	0.08		
**Dependent variable: CPT-II Omissions**							
Step 1						0.02	0.02
Constant	47.71	40.76	54.66	3.50			
Neurological Predictor Scale	0.81	–0.54	2.17	0.68	0.13		
Step 2						0.06	0.04
Constant	43.93	35.98	51.89	4.00			
Neurological Predictor Scale	1.10	–0.27	2.47	0.69	0.18		
CBCL Sleep Composite-6	1.28	–0.09	2.65	0.69	0.21		
**Dependent variable: CPT-II Hit RT**							
Step 1						0.06	0.06
Constant	45.33	37.82	52.84	3.77			
Neurological Predictor Scale	1.65	0.19	3.10	0.73	0.25		
Step 2						0.04	0.00
Constant	45.93	37.07	54.78	4.44			
Neurological Predictor Scale	1.60	0.09	3.11	0.76	0.24		
CBCL Sleep Composite-6	–0.19	–1.70	1.31	0.76	–0.03		
**Dependent variable: CPT-II Commissions**							
Step 1						0.00	0.00
Constant	51.25	45.34	57.16	2.97			
Neurological Predictor Scale	–0.33	–1.48	0.82	0.58	–0.06		
Step 2							
Constant	51.22	44.31	58.13	3.47		0.00	0.00
Neurological Predictor Scale	–0.33	–1.52	0.86	0.60	–0.06		
CBCL Sleep Composite-6	0.01	–1.18	1.12	0.60	0.00		

*CI, confidence interval; LL, lower limit; UL, upper limit.*

*p < 0.05; **p < 0.01; ***p ≤0.001.

Subsequently, secondary regression analyses were performed on cognitive dimensions that are vulnerable to sleep difficulties. For the regression analysis with CBCL Attention Problems as the dependent variable, Model 1 indicated that the variance accounted for (*R*^2^) by the control variable (disease burden) equaled 0.09, which was significantly different from zero, *F*(1,81) = 8.41, *p* < 0.01. In Model 2, the change accounted for by the CBCL Sleep Composite-6 (Δ*R*^2^) was equal to 0.19, which was significantly different from zero, *F*(1,80) = 20.53, *p* < 0.001. Thus, 19% of the variance in CBCL Attention Problems was accounted for by the CBCL Sleep Composite-6 score. None of the performance-based measures of cognition was significantly associated with sleep disturbance after adjusting for overall disease burden [i.e., DSF: Δ*R*^2^ = 0.00, *F*(1,77) = 0.06, *p* = 0.81; DSB: Δ*R*^2^ = 0.01, *F*(1,77) = 0.52, *p* = 0.47; CPT-II Omissions: Δ*R*^2^ = 0.04, *F*(1,79) = 3.46, *p* = 0.07; CPT-II Hit RT: Δ*R*^2^ = 0.00, *F*(1,78) = 0.07, *p* = 0.80; CPT-II Commissions: Δ*R*^2^ = 0.00, *F*(1,79) = 0.00, *p* = 0.99]. Notably, results of all regression analyses were comparable when performed without inclusion of a control variable. Furthermore, exploratory interaction effects were not observed for gender, disease burden, and months off treatment, except for a significant interaction for months off treatment and CBCL Sluggish Cognitive Tempo and CBCL Attention Problems. Specifically, for both variables, the association with the CBCL Sleep Composite was strongest for participants who completed treatment most recently (CBCL Sluggish Cognitive Tempo: *R*^2^ = 0.45; CBCL Attention Problems: *R^2^*= 0.37), and weakest for children with the longest time since completion of treatment (CBCL Sluggish Cognitive Tempo: *R*2 = 0.12; CBCL Attention Problems: *R*^2^ = 0.13).

## Discussion

The primary aim of the current study was to investigate whether a higher level of sleep disturbance was associated with worse sluggish cognitive tempo (SCT) and/or cognitive functions associated with SCT—including attention and working memory—in a clinical sample of PBT survivors. To our knowledge, this is the first study to investigate the relationship between SCT and sleep in the PBT survivor population. The analysis investigating the primary aim provided support for the hypothesized relationship between a higher level of sleep disturbance and worse SCT. The secondary analyses focusing on aspects of attention and working memory were partially supported. Specifically, while no significant association was observed for Digit Span Forward, Digit Span Backward, CPT-II Omissions, CPT-II Hit RT, or CPT-II Commissions higher levels of sleep disturbance were related to worse CBCL Attention Problems.

In terms of overall frequency of sleep difficulties, nearly a third of caregivers reported at least one significant sleep disturbance in their child, and over two-thirds of caregivers reported at least occasional occurrence of sleep disturbances. These results align with what is known for PBT survivors, with estimates of sleep problems ranging between 20 and 82% (Brimeyer et al., 2016; Pilotto et al., 2018; van Kooten et al., 2019), as compared to rates of 12–25% in healthy populations (Melendres et al., 2004; van Litsenburg et al., 2010). Somnolence and increased sleep duration were the most frequently endorsed individual sleep-related symptoms, which parallels prior literature suggesting that excessive daytime sleepiness is the most common sleep-related disturbance in children diagnosed with cancer (Rosen and Brand, 2011; Walter et al., 2015).

Comparison of sample performance to normative means indicated that our sample performed significantly worse than the normative mean for all variables except CPT-II Omissions and CPT-II Commissions, which largely validates that our sample conformed to known neurocognitive vulnerabilities in PBT survivors. Among all included cognitive variables, caregiver ratings of SCT were most frequently impaired (30%; <1.5 *SD* relative to the mean); in contrast, attention problems were rated as impaired by 20.48% of caregivers. The relatively high endorsement of SCT in the current study adds to mounting evidence that SCT is common in the PBT population in a manner that is distinct from other neurocognitive deficits (Kahalley et al., 2011; Willard et al., 2013; Peterson et al., 2021).

Our findings suggest that the relationship between SCT and sleep disturbance observed in pediatric populations with neurodevelopmental conditions such as ADHD and autism (e.g., Mayes et al., 2021; Fredrick et al., 2022) appears applicable to PBT survivors as well, and this has implications for screening and assessment. Recognition of this association underscores the importance of differential assessment of SCT and sleep to improve identification of core difficulties experienced by PBT survivors with the goal of maximizing HRQOL. For example, recent evidence suggests that SCT is associated with social difficulties such as low social engagement and initiation, along with withdrawal and isolation (Becker et al., 2019), and interestingly the nature of these social difficulties is similar to that of the social competence difficulties commonly experienced by PBT survivors (Hocking et al., 2015). Similarly, there is overlap between SCT and anxiety/depression symptoms, excessive daytime sleepiness, and low motivation (Smith et al., 2019), which also may be within the scope of HRQOL surveillance for PBT survivors. Findings of the current study suggest that when only focusing on clinic-based measures of cognition administered one-on-one with a patient, the relationship between sleep and cognitive functioning may be missed despite being potentially meaningful for daily functioning.

Thus, incorporating assessment of both sleep and SCT into existing psychological and neuropsychological monitoring of PBT survivors might contribute to clearer differentiation of symptoms and thereby improve linkage to targeted intervention. Indeed, interventions for varied difficulties ranging from sleep to apathy to attention problems to SCT would likely involve markedly different treatment approaches. For example, there may be medication options that could be researched in PBT survivors demonstrating SCT, such as modafinil (Kumar, 2008), which has demonstrated efficacy for symptoms including attention, speed, and drowsiness in adult cancer patients (Lundorff et al., 2009). Sleep interventions might look quite different, as parents tend to be more receptive to behavioral sleep interventions compared to medication options (Daniel et al., 2016). There is also the possibility that improvement of sleep and/or SCT symptoms could enhance response to other supports—ranging from social to academic interventions—by reducing the impact of an overarching problem that is not specific to any neurocognitive domain. For example, directing intervention efforts at sleep and/or SCT could possibly enhance cognitive training interventions, which have thus far provided limited generalizable relief to PBT survivors experiencing significant neurocognitive late effects (Hocking et al., 2021). To illustrate, in a sample of healthy adolescents, treatment of sleep disturbance has been shown to result in improved executive functioning (de Bruin et al., 2015), though the extent to which such findings generalize to PBT survivors is unknown, given the impact of the tumor, complications, and treatment on the cognitive developmental trajectory in this population (Mahajan et al., 2021). Results of our archival study do not address whether there is a causal relationship between sleep and SCT, so further research will be necessary to identify optimal treatment paradigms to target core problems that may manifest as overlapping symptoms and difficulties.

The fact that parent-reported sleep was associated with SCT and parent-rated attention problems, but not with individually administered clinic-based measures of attention of working memory, is important to consider. One possible explanation for the discrepant findings between parent-rated and performance-based measures of cognition is that these modalities may tap into different aspects of cognitive performance. Specifically, Toplak et al. (2013) suggested that a performance-based measure assesses whether someone is able to successfully complete an executive functioning task in isolation, while not measuring the extent to which that individual is able to regulate their behavior in the absence of continuing direction by an examiner and/or in the presence of multiple competing stimuli and distractions. Thus, it may be that sleep disturbances—at least with respect to the relatively mild levels reported in the present study—impact cognitive functioning in everyday life more than performance on discrete tests. When combined with performance-based measures, collateral (e.g., parent, teacher) ratings may provide a more complete picture of a PBT survivor’s capacity for cognitive functioning as well as their efficacy at implementing those cognitive functions in everyday life (Toplak et al., 2013). Therefore, including ratings of SCT and attention problems in neuropsychological evaluations may enhance detection of subtle cognitive difficulties associated with sleep disturbance.

The idea that relatively mild sleep disturbances are related more to the execution of cognitive tasks in less structured settings than to test performance in an evaluation setting is in keeping with the model proposed by Monk (2012), which suggests that chronic sleep disruption can lead to a progressive loss of alertness and motivation as time progresses. Supporting this idea, several studies have demonstrated associations between various types of sleep disruption and lower engagement in tasks that are perceived as more cognitively demanding (Engle-Friedman et al., 2010, 2018; Libedinsky et al., 2013). Further, the impact of motivation on performance in PBT survivors was illustrated by Holland et al. (2016), who demonstrated that external incentive improves effort on discrete timed academic tasks, indicating that motivation is a fluctuating variable that can substantively alter the situational performance of PBT survivors. Considering these findings, it may be that PBT survivors who experience sleep difficulties may choose less effortful activities when possible, and this choice may be available to a lesser extent during a neuropsychological evaluation as compared to daily life. This conceptualization would align with the known and persistent difficulty with the execution of cognitive tasks in daily life experienced by PBT survivors in everyday life, even when corresponding aspects of clinic-based assessment reflect average overall performance relative to the general population (Holland et al., 2015). Notably, our findings align with the notion that the construct of SCT—among other concepts—taps into a motivational component of cognitive functioning in daily life (Becker et al., 2016; Smith et al., 2019).

## Limitations and Future Directions

The findings of this study should be considered in light of a number of limitations, foremost of which being the method of assessing sleep disturbances, which did not include objective measures such activity tracking or polysomnography. While the CBCL Sleep Composite has been validated against accepted sleep measures and has been deemed appropriate for archival studies (Gregory et al., 2011; Becker et al., 2015a,b; Murray et al., 2016) objective characterization and differentiation of specific types of sleep disturbances may help to clarify the nature of the relationship between sleep and cognition in PBT survivors. In other words, the present study provides evidence for an association between sleep and cognition in this population, but the specific elements of sleep disturbance that may have relatively more or less impact on cognitive performance require further elucidation.

Another limitation of the CBCL Composite is a lack of clarity regarding the timeframe during which parents are rating sleep disturbances. Parents are instructed to rate their child’s behavior over the past 6 months, and this leaves unclear the extent to which the presence or absence of sleep disruptions directly prior to the time of their ratings may have affected their reports as well as association with cognitive tests completed at the same time as the ratings. Thus, our study neglects the important consideration of whether there is a differential effect on cognition for longer-term chronic sleep disturbances as compared to more acute sleep problems. In addition to caregiver ratings, when feasible, self-reports of sleep may provide valuable information. For instance, Short et al. (2013) observed a tendency for parents to underreport sleep problems in their adolescent children. Thus, the level of sleep disturbance experienced by at least some of our study participants may have been higher than suggested by caregiver ratings. Finally, the possibility that the association between parent-rated sleep and parent-rated cognition partially reflects rater bias cannot be excluded. Specifically, while a suggested advantage of ratings relative to objective cognitive measures is that they may tap more directly into real-word circumstances, it has also been stressed that ratings may be impacted by a halo-effect, central tendency bias, social desirability bias, or a general propensity to under- or over-report (Toplak et al., 2013; Dekker et al., 2017; Emser et al., 2018; Brandt et al., 2021). For instance, parental anxiety may increase attention for the child’s symptoms, which, in turn, may affect their ratings (Smith et al., 2020).

Characterization of risks for sleep disturbances in PBT survivors remains incomplete. Thus, it is important to further investigate biological [e.g., tumor-related factors such as tumor type and location (Helligsoe et al., 2022) or treatment modalities], environmental, family-related, psychological, and behavioral factors that are most strongly associated with the onset and perpetuation of sleep problems in PBT survivors. The construct and measurement of SCT is evolving (Becker et al., 2019), and as measurement methods advance future researchers can incorporate more refined metrics to assess SCT in PBT survivors. Additionally, future researchers may use prospective designs at each stage of the trajectory from diagnosis to active treatment to long term PBT survivorship to determine whether sleep is causally related to cognitive performance, as appears to be the case in other types of cancer. More comprehensive understanding of the nature of the association between sleep and cognitive performance in PBT survivors may yield insights into important targets for prevention and intervention that could have a significant positive impact on aspects of health-related quality of life.

## Conclusion

In the present sample of PBT survivors, higher levels of parent-rated sleep disturbances were associated with higher levels of sluggish cognitive tempo (SCT) and worse parent-reported attention functioning. There were no significant correlations with performance-based measures of attention and working memory. Findings of the current study highlight the importance of further investigation into the relationship between sleep and cognition in this vulnerable population, as greater knowledge in this regard may assist efforts to maximize cognitive outcome and HRQOL in PBT survivors. The current study additionally suggests further investigation of SCT in this population is warranted, as it may be more sensitive to detecting possible associations with sleep disturbance relative to discrete measures that assess cognitive performance under ideal circumstances.

## Data Availability Statement

The raw data supporting the conclusions of this article will be made available by the authors, without undue reservation.

## Ethics Statement

The studies involving human participants were reviewed and approved by Institutional Review Board, University of Texas MD Anderson Cancer Center. Written informed consent from the participants’ legal guardian/next of kin was not required to participate in this study in accordance with the national legislation and the institutional requirements.

## Author Contributions

IO: review and editing, original draft, conceptualization, formal analysis, and visualization. AH, RH, and DH: review and editing and supervision. AC: review and editing, formal analysis, and supervision. WZ, MB, and GY: review and editing and investigation. PS: review and editing, original draft, supervision, conceptualization, resources, data curation, and visualization. All authors approved the submitted version.

## Conflict of Interest

The authors declare that the research was conducted in the absence of any commercial or financial relationships that could be construed as a potential conflict of interest.

## Publisher’s Note

All claims expressed in this article are solely those of the authors and do not necessarily represent those of their affiliated organizations, or those of the publisher, the editors and the reviewers. Any product that may be evaluated in this article, or claim that may be made by its manufacturer, is not guaranteed or endorsed by the publisher.

## Abbreviations

CBCL: Child Behavior Checklist
CPT-II: Conners Continuous Performance TestSecond Edition
DSF: Digit Span Forward
DSB: Digit Span Backward
PBT: pediatric brain tumor
GAI: general ability index
HRQOL: health-related quality of life
Hit RT: Hit Reaction Time
NPS: Neurological Predictor Scale
SES: socioeconomic status
SCT: sluggish cognitive tempo
WAIS-IV: Wechsler Adult Intelligence Scale–Fourth Edition
WISC-IV/V: Wechsler Intelligence Scale for Children–Fourth Edition/Fifth Edition.
